# Nitrogen Limitation Promotes Accumulation and Suppresses Release of Cylindrospermopsins in Cells of *Aphanizomenon* Sp.

**DOI:** 10.3390/toxins6102932

**Published:** 2014-09-30

**Authors:** Karina Preußel, Ingrid Chorus, Jutta Fastner

**Affiliations:** Federal Environment Agency, Schichauweg 58, 12307 Berlin, Germany; E-Mails: karina.preussel@gmx.de (K.P.); ingrid.chorus@uba.de (I.C.)

**Keywords:** *Aphanizomenon*, cylindrospermopsin, cyanobacteria, deoxy-cylindrospermopsin, release, production

## Abstract

As the biosynthesis of cylindrospermopsin (CYN) is assumed to depend on nitrogen availability, this study investigated the impact of nitrogen availability on intra- and extracellular CYN and deoxy-CYN (D-CYN) contents in three *Aphanizomenon* strains from temperate waters. Nitrogen deficient (−N) cultures showed a prolonged growth phase and intracellular toxin accumulation by a factor of 2–6. In contrast, cultures with additional nitrate supply (+N) did not accumulate CYN within the cells. Instead, the maximum conceivable CYN release estimated for dead cells (identified by SYTOX^®^ Green staining) was much lower than the concentrations of dissolved CYN actually observed, suggesting these cultures actively release CYN from intact cells. Furthermore, we found remarkably altered proportions of CYN to D-CYN: as batch cultures grew, the proportion of D-CYN increased by up to 40% in +N medium, whereas D-CYN remained constant or decreased slightly in −N medium. Since +N cultures showed similar toxin patterns as −P cultures with increased extracellular CYNs and higher proportion of D-CYN we conclude that nitrogen limitation may affect the way the cells economize resources, especially the yield from phosphorus pools, and that this has an impact on CYN production and release. For water management, these result imply that nutrient availability not only determines the abundance of potentially CYN-producing cyanobacteria, but also the amount of extracellular CYNs (challenging drinking-water treatment) as well as the ratio of D-CYN to CYN (affecting toxicity).

## 1. Introduction

The cyanobacterial toxin cylindrospermopsin (CYN) has been found worldwide [1,2] with frequent occurrence in sometimes potentially hazardous concentrations particularly in tropical and subtropical waters [3]. Screening programs have shown its widespread occurrence also in European water bodies where CYN is mainly produced by *Aphanizomenon*, and possibly also by *Anabaena* [4,5,6,7]. Data available so far suggest CYN concentrations to usually be well below 10 µg/L in Central Europe with the highest concentration reported to date of 126 µg/L found in Italy [8,9]. However, management efforts to reduce nitrogen concentration in water bodies may potentially increase the frequency and concentrations of CYN-producing cyanobacteria since they belong to the Nostocales, which are able to fix atmospheric nitrogen and can thus compensate nitrogen deficiency [10,11]. Moreover, prolonged growth periods caused by climate change are proposed as mechanism increasing the occurrence of potentially toxic Nostocales [12].

Beside CYN two other congeners, 7-epi-cylindrospermopsin and 7-deoxy-cylindrospermopsin (D-CYN), exist and have been observed in a wide range of CYN-producing cyanobacteria, e.g., *Aphanizomenon ovalisporum*, *Raphidiopsis curvata* and *C. raciborskii*. [1,13,14,15]. Data on their occurrence and persistence/degradation in the field are hardly reported to date.

As previously found in tropical systems, a high share of CYN is present as extracellular fraction also in temperate waters. Rücker *et al.* [8] reported that in 31% of the samples from German lakes more than 80% of total CYN was extracellular. Due to its chemical stability and poor degradation in the water, CYN is highly persistent in many lakes [16,17,18].

In contrast to the cyanobacterial hepatotoxic peptide microcystin, which is scarcely found extracellularly and appears to be released only by dead or lysed cells [19], extracellular CYN and D-CYN so far has always be detected in media of exponentially growing populations [15,20,21], suggesting that CYNs are released into external medium by cells. However, highest extracellular CYNs concentrations were found in older blooms [16] and in the stationary phase of batch cultures [15,20,22], implying that release increases with senescence of cells.

As the toxicity of CYN mainly derives from its property to inhibit eukaryotic protein synthesis [23], the metabolite may affect various aquatic organisms as well as human health [1,24,25,26]. The extracellular fraction could act directly without a pathway via consumption by organisms (including on aquatic plants). Moreover, persistent extracellular CYN in surface waters used as drinking water resource has important consequences for treatment procedures because conventional flocculation/filtration processes are not effective for removing dissolved toxins [24,27].

The putative biosynthetic pathway of CYN is described as integrated PKS/NRPS system for several genera of Nostocales and Oscillatoriales [28,29,30]. The CYN-biosynthesis gene cluster is flanked by gene sequences which are homologous to sequences found in *Nostoc*. Their transcription is directly regulated by the global nitrogen regulator (NtcA) in *Nostoc*—an activator of nitrogen assimilation genes. As no obvious promoter region could be identified within the *CYN* gene cluster, the simultaneous regulation of CYN synthesis by NctA would be plausible. A further possible regulation of CYN synthesis by a protein which specifically binds to a DNA fragment of the synthetase gene cluster was discussed for *Aphanizomenon ovalisporum* [31]. Homologues of that protein (antibiotic resistance protein B) are known to be transcription factors and are implicated in the regulation of genes during acclimation to nutrient depletion and in transition to stationary phase.

Indeed, the most pronounced changes of CYN production were observed in batch cultures at the beginning stationary growth phase. Saker and Griffith [32] found nearly constant intracellular toxin contents per unit biovolume during growth in the exponential phase of *C. raciborskii* strains, but a rapid increase up to 2–3-fold values at the beginning stationary phase. An impact of nitrogen availability was observed in several Australian *C. raciborskii* strains with highest intracellular CYN contents in batch cultures free of nitrogen and intracellular CYN contents (per unit dry weight) varying at maximum by a factor of 2 [33]. In contrast, CYN content in *Aphanizomenon ovalisporum* was lower in cultures free of nitrogen compared to growth under combined nitrogen supply [34].

Furthermore, all batch culture studies which included the analysis of extracellular CYN [20,22,33] report an extreme and rapid increase of extracellular CYN concentrations up to 50% of total CYN in stationary phase cultures, which could not be explained purely by release from lysed cells.

Based on our former observations of an assumed active CYN release under light and temperature conditions probably causing physiological stress [21] and the proposed nitrogen dependent biosynthesis, we investigated the influence of nitrogen availability on the production and release of cylindrospermopsins in three strains of indigenous *Aphanizomenon* sp.; We performed additional experiments under phosphorus limitation to test the hypothesis of a possible interaction between cellular nutrient pools of nitrogen and phosphorus which might affect CYN biosynthesis or release.

Our results may contribute to assess future trends of CYNs concentrations in temperate lakes in the light of ongoing efforts to reduce nutrient concentrations in catchments and water-bodies.

## 2. Results and Discussion

### 2.1. Results

#### 2.1.1. Influence of Nitrogen Supply

**Figure 1:**
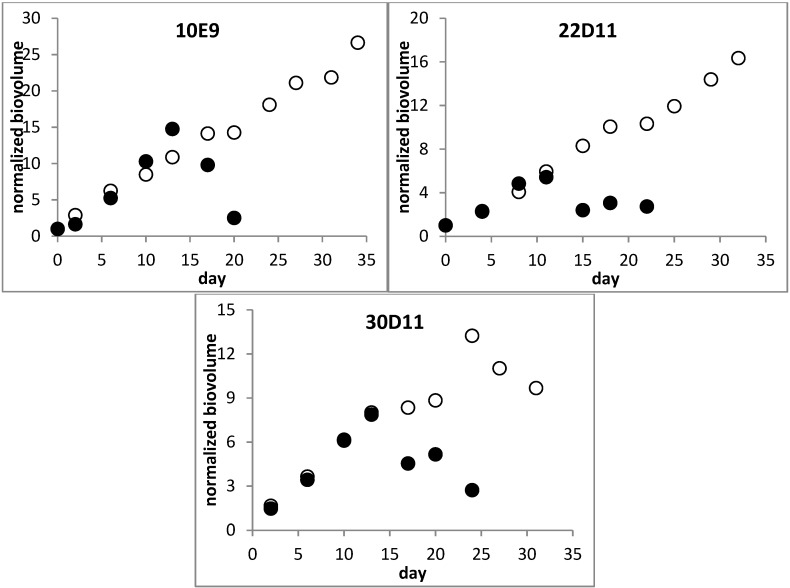
Growth of three *Aphanizomenon* sp. strains in +N (●) and −N (○) batch cultures. Biovolume concentrations are normalized to the biovolume at the beginning of the culture (value at day 0 = 1) for direct comparability. Real concentrations at day 0 are given in Table 1.

The transfer of *Aphanizomenon* sp. cells from +N into −N nutrient solution prolonged growth, promoted internal accumulation of CYNs and reduced toxin release as compared to growth in +N medium: Growth of +N batch cultures dropped after 11–13 days, while −N cultures showed further increase of intact biomass up to the end of the experiment (day 35), respectively, up to day 25 for strain 30D11 (Figure 1).

**Figure 2:**
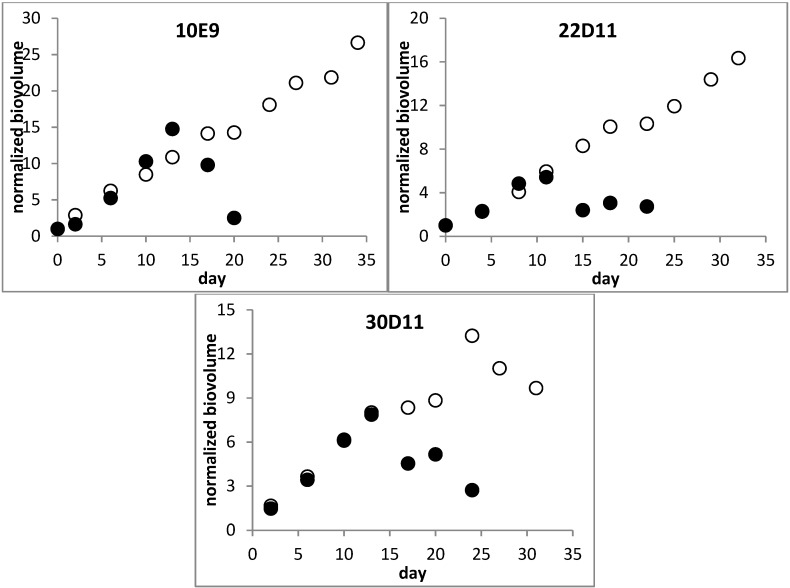
Total cylindrospermopsins concentrations in +N (♦) and −N (◊) batch cultures of three *Aphanizomenon* sp. strains and relative proportion of the precursor metabolite D-CYN (+N: ▲ and −N: ∆). CYNs concentrations are normalized to the concentrations at the beginning of the culture (value at day 0 = 1) for direct comparability. Real CYNs concentrations at starting points are given in Table 1.

**Figure 3:**
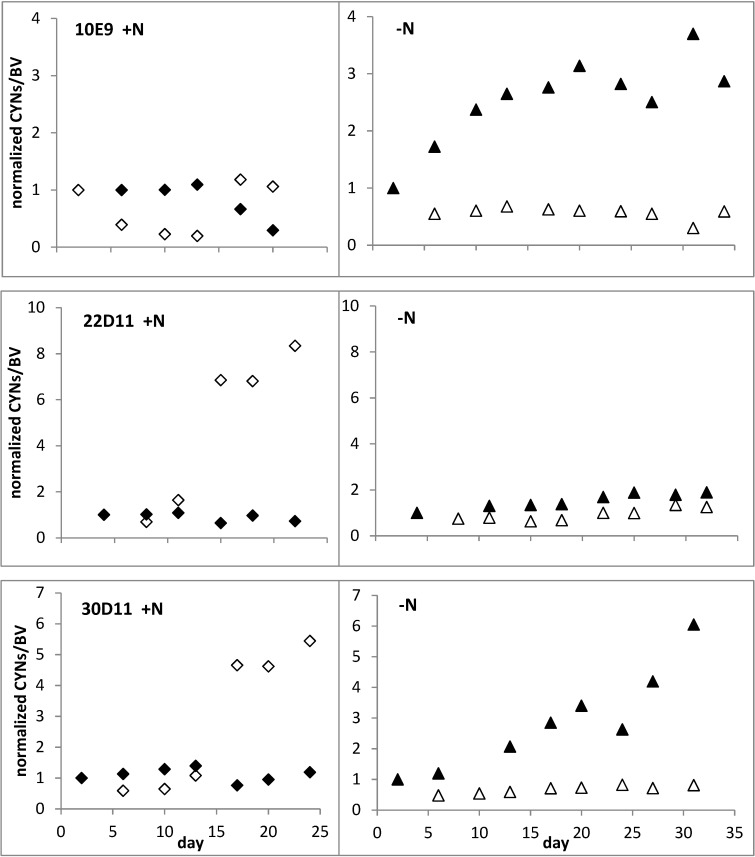
Biovolume related intracellular (solid symbols) and extracellular (open symbols) contents of total cylindrospermopsins (CYNs) of +N (♦, ◊) and −N (▲, ∆) in batch cultures. Contents are given as values normalized to the contents at the beginning of the culture (value at day 0 = 1) for direct comparability. Real biovolume related CYNs contents at the beginning of each batch culture experiment are given in Table 1.

As long as growth of both −N and +N cultures was similar (*i.e*., until day 11–13), the total toxin concentrations (sum of intra- and extracellular CYNs) between the different treatments and between strains were nearly identical (Figure 1 and Figure 2). In contrast, substantial differences were found for the intracellular and the extracellular toxin fraction (Figure 3): during log-phase growth the intracellular toxin content of the cells from +N cultures remained quite constant. Subsequently, in the phase of culture decline (of all three strains in the +N experiments) intracellular content stayed constant or decreased, while extracellular concentrations continued to increase. However, the −N cultures showed a continuous increase of the intracellular toxin content during their growth, up to 2–6 fold of the starting concentration. Their extracellular toxin concentration did not increase over time; moreover it was much lower in all −N cultures as compared to the corresponding +N cultures (Figure 3).

The nitrogen supply also affected the proportion of the minor toxin component D-CYN to CYN (Figure 2). In +N cultures, the share of D-CYN increased during growth in both the intracellular and the extracellular fraction, whereas it decreased remarkably and to a similar extent in both fractions of −N cultures (data for the separate fractions are not shown).

**Table 1:** Biovolumes and cylindrospermopsins per unit biovolume at the beginning of the batch cultures (day 0).

Strains	10E9		22D11		30D11	
**Conitions**	**+N**	**−N**	**+N**	**−N**	**+N**	**−N**
Biovolume (mm^3^ L^−1^)	25.5	25.5	17.9	17.9	75.1	75.1
CYNs_in_ (ng mm^−3^)	386.4	289.6	390.3	341.9	801.0	751.0
CYNs_ex_ (ng mm^−3^)	80.8	24.9	17.9	24.2	22.1	23.6

CYNs_in_—intracellular cylindrospermopsins; CYNs_ex_—extracellular cylindrospermopsins.

#### 2.1.2. Influence of Phosphorus Supply

Adaptation to the reduced growth rate in consequence of the reduced supply of inorganic phosphorus influenced the release of CYNs in all three strains (Table 2). All strains showed higher extracellular concentration of CYNs under −P conditions. Moreover, the BV related contents of CYNs (sum of all fractions) were higher in −P cultures of 30D11 and 10E9 as compared to the respective +P variant, while they were similar for strain 22D11. A clear increase of D-CYN was observed under −P, especially in the extracellular fraction (Table 2).

**Table 2:** Effect of phosphorus on growth rate and toxin contents of semi-continuous cultures of different *Aphanizomenon* sp. strains.

Strains	Conditions	µ ^a^ (d^−1^)	CYN_in_^b^ (ng mm^−3^)	D-CYN_in_^b^ (ng mm^−3^)	CYN_ex_^c^ (ng mm^−3^)	D-CYN_ex_^c^ (ng mm^−3^)	D-CYN ^d^ (%)
**10E9**	+P	0.229 ± 0.027	431 ± 54	36 ± 8	78 ± 8	8 ± 2	8 ± 2
	−P	0.122 ± 0.023	313 ± 38	44 ± 11	286 ± 49	81 ± 23	17 ± 3
**22D11**	+P	0.247 ± 0.032	339 ± 26	87 ± 7	29 ± 3	11 ± 1	21 ± 2
	−P	0.133 ± 0.102	8 ± 2	4 ± 2	219 ± 85	172 ± 68	44 ± 1
**30D11**	+P	0.231 ± 0.054	559 ± 82	56 ± 18	151 ± 135	31 ±33	10 ± 2
	−P	0.125 ± 0.040	589 ± 114	105 ± 22	320 ± 83	80 ± 24	17 ± 2

All values are means of 10 samples with confidence limits for *p* = 0.05; ^a^ Growth rate (d^−1^); ^b^ Biovolume related intracellular toxin contents; ^c^ Biovolume related extracellular toxin contents; ^d^ Share of total cylindrospermopsins.

#### 2.1.3. Toxin Release

**Figure 4:**
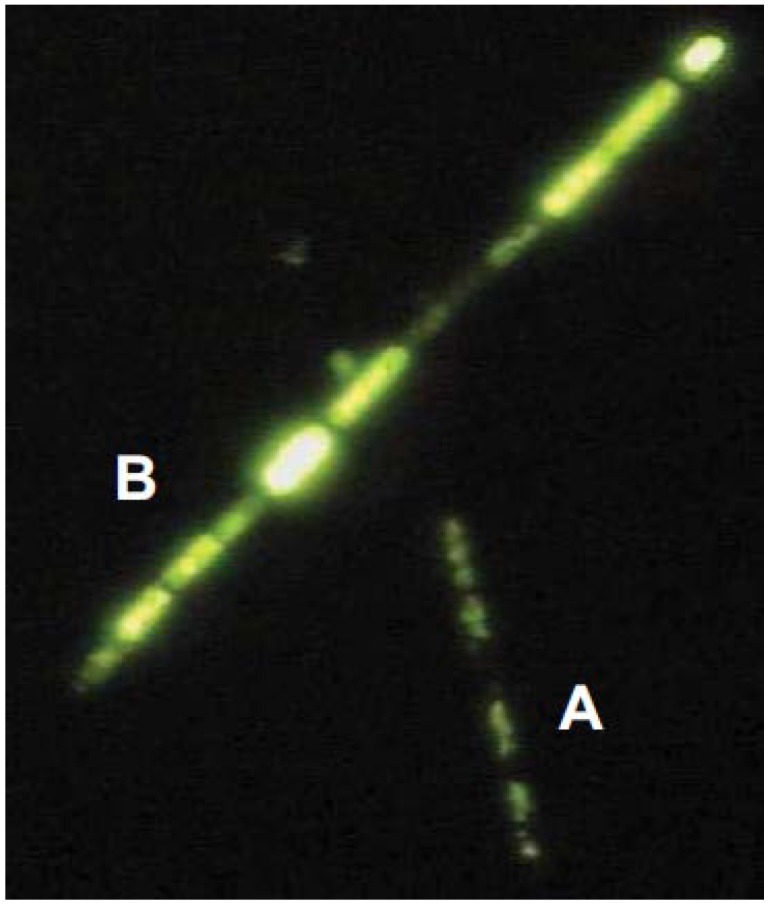
Filaments of strain 22D11 stained with SYTOX^®^ Green—cells with intact membrane (**A**) with basic fluorescence and cells with increased membrane permeability; (**B**) clearly enhanced fluorescence signals.

Staining cultures with SYTOX^®^ Green allowed the discrimination of filaments and filament segments with intact cell membrane from those with impaired (and therefore permeable) membranes (Figure 4).

The biovolume (BV) of cells with permeable membranes was used to calculate a maximum expected amount of extracellular CYNs which could be released from lysing cells in +N and −N cultures by assuming that all of the damaged cells at day *X* had been damaged since the previous sampling day (*X* − 1) as follows (1) with index *X* for the corresponding sampling day:

(1) $$\text{Maximum Expected Extracellular}{\text{CYNs}}_{X}=\;\overset{n}{\underset{X=1}{\sum }}\text{damaged}\;{\text{BV}}_{X}\;\times \text{intracellular}\;{\text{CYNs}}_{X-1}$$

**Figure 5:**
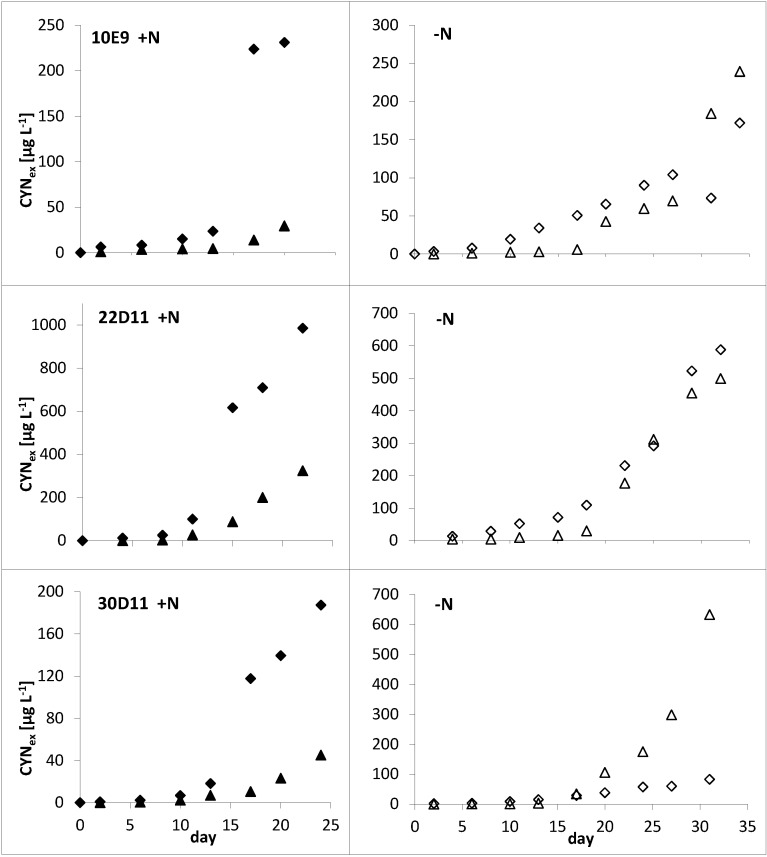
Real (♦,◊) and expected (▲,∆) concentrations of extracellular CYNs in culture medium of +N and −N batch cultures of *Aphanizomenon* sp. strains.

Hence, the expected concentrations represent maximum values of extracellular CYNs, assuming continuous lysis of filaments as well as accumulation of all released toxin in the culture without any biodegradation (an extreme but possible scenario due to the poor biodegradability of CYN). A key result is that actually detected concentrations of extracellular CYNs in all +N-cultures were substantially higher than those expected on the basis of the observed cell damage and the maximum release/accumulation scenario described above (Figure 5). In contrast, extracellular concentrations in −N cultures corresponded to the expected maximum values (strains 10E9 and 22D11) or were somewhat lower (strain 30D11). This suggests that CYN is actively released when growth is not limited by N.

The ratio of dead to viable cells has not been determined for the P experiments with semi-continuous cultures, as under steady state conditions there is no reason to assume that this ratio would change.

### 2.2. Discussion

We studied the nitrogen-dependent growth and toxin production of *Aphanizomenon* sp. strains in batch cultures—an approach which integrates the processes of progressive depletion of macro- and micro-nutrients during growth and biomass accumulation. These experiments showed surprising results, chiefly the prolonged exponential growth of the nitrogen-limited (−N) cultures and differences between +N and −N cultures in the proportions of intra- to extracellular CYN as well as of D-CYN to CYN. While −N and +N cultures produced similar amounts of CYNs during their exponential growth phase, cells in −N cultures contained at least two times higher total CYNs at the end of the experiment. Our results contrast with findings for *Aphanizomenon ovalisporum* where the CYN content first decreased and then increased during exponential growth in cultures free of nitrogen, but never reached the content of cultures grown under combined nitrogen conditions [34]. One reason for this discrepancy could be that this study had not considered the extracellular CYN fraction, which can amount to 20%−50% on total CYN depending on strain and growth phase, as shown for *Aphanizomenon ovalisporum* strains as well as *C. raciborskii* [15,35].

Our data suggest that nitrogen limiting conditions promote prolonged growth, possibly by changed metabolic activities and efficiency of resource consumption. Indeed, 12% of all genes in *Cylindrospermopsis raciborskii* CS-505 altered their expression within 24 h of N-deprivation [36]. These up- or down-regulated genes are not only involved in the formation of heterocysts and N2 fixation, but also in inorganic carbon acquisition/storage, cell division and phycobilisome synthesis/degradation. In consequence of these results, such nutrient dependent gene regulation is a worthwhile focus for future experiments to identify key processes responsible for population development under changing nutrient availability in freshwaters.

Furthermore, our results indicate the existence of a regulated transport of CYNs from intact cells into water/medium: The discrepancy between expected and actually detected concentrations of extracellular CYNs in +N cultures can only be explained by export of CYNs from intact cells. This is supported by the assumption of a CYN-transporter which has been deduced from the biosynthesis cluster CyrK [29,30]. To our knowledge our results are the first direct indication that a large amount of extracellular CYNs originates from intact cells.

In our experiments, the release of CYNs from intact cells was apparently regulated by nutrient availability. We showed that two processes caused the accumulation of extracellular CYNs in +N cultures—a passive release from lysed cells and an additional release from intact cells. In contrast, the amount of extracellular CYNs in −N cultures could be explained by release from damaged cells alone, and thus, under nitrogen-limiting conditions an export of CYNs from intact cells seems to be reduced or completely shut down. This was also reflected by the increase of internal CYNs contents.

Interestingly, the increase of intracellular CYNs contents (respectively, the reduced CYNs export) coincided with a prolonged growth of the strains. In contrast, the assumed active release in the +N batch cultures coincides with the decline of growth. Increased release of CYN was also already found in phosphorus limited cultures of *Aphanizomenon ovalisporum* [37]. These experiments showed that the extracellular CYN induced substantial excretion of alkaline phosphatase from other planktonic organisms into water and thereby enhanced the phosphorus supply for *Aphanizomenon*. This result is coherent with Rzymski *et al.* [38] who show that CYN but also spent medium from *C. raciborskii* induced the up-regulation of alkaline phosphatase in *Microcystis* cultures [38]. The observed co-occurrence of CYN release and growth impairment in our experiments suggested that the cells also were P-limited at this point of time in the +N-cultures. Since the cellular P was not determined, we conducted additional experiments to study the influence of phosphorus on growth and CYN/D-CYN production and release.

The results of our experiments with the same strains under pronounced phosphorus limitation support the assumption that the +N cultures reached phosphorus limitation earlier than −N cultures (Table 2). All patterns observed for the −P cultures were similar to the results from +N cultures. The increased release of CYNs and the increasing ratios of D-CYN/CYN (which are associated with the stationary growth phase in +N batch cultures) were also found in −P cultures.

Therefore, taking all these results together, we suggest that −N conditions may alter resource economizing—especially the yield from available phosphorus pools, and thus also have an impact on CYN production and release. The results were consistent for three different strains, suggesting that they may be more widely valid for indigenous *Aphanizomenon flos-aquae*/*gracile*.

In consequence for field populations, our results suggest that a further reduction of nitrogen concentrations in water bodies could favour overall higher CYNs concentrations in *Aphanizomenon*-dominated lakes by two mechanisms: by their competitive advantage due to their ability to fix atmospheric nitrogen under nitrogen limitation per se, and by the ability of *Aphanizomenon*-populations to grow longer and thus reach a higher final biomasses than under nitrogen rich conditions. In such scenarios rapidly increasing concentrations of extracellular CYNs could be expected after population breakdown due to the remarkable intracellular enrichment of the toxins during the growth period. Indeed, extracellular CYN concentrations often peak after intracellular concentrations and then are substantially higher than these [39].

Our results regarding an increased proportion of D-CYN (up to 40%) during the stationary phase of +N cultures is in line with that of Davis *et al.* [15] for nitrogen repleted cultures of *C. raciborskii*. In our study, an increase of D-CYN was equally observed in −P cultures. Assuming that +N cultures are likely to become P-limited in the stationary phase, this increase may be explained by an influence of phosphorus limitation on the hydroxylation of D-CYN. A next step would be to conduct expression experiments to investigate whether the last step of CYN biosynthesis might be decelerated by reduced translation of CyrI and synthesis of the 2-oxo-glutarate-dependent iron oxygenase, or whether it is rather due to substrate inhibition of the enzyme [40].

D-CYN occurs in many CYN-producing *Aphanizomenon*, *C. raciborskii* and *Raphidiopsis* [41,42], and an increase in its share with culture age has been shown in this study and in a study by Davis *et al.* [15]. While D-CYN was found non-toxic to mice by intraperitoneal injection, *in vitro* it inhibits protein synthesis with potency similar to that of CYN [43,44]. A more comprehensive understanding of the toxicity of this compound is necessary in order to clarify whether the observed shifts in the ratio of D-CYN to CYN are relevant for risk assessment.

## 3. Experimental Section

### 3.1. Cyanobacterial Strains

The non-axenic strains used in this study were isolated in 2004 from Melangsee (strain 10E9), Heiliger See (strain 22D11) and Petersdorfer See (strain 30D11), all situated in the region of Brandenburg, Germany.

Strains 10E9 and 22D11 were isolated as *Aphanizomenon flos-aquae* [4] but changed their morphology in culture, developing morphological features of both *Aph. flos-aquae* and *Aph. gracile*. All three strains could not clearly be referred to one of the species, neither on the basis of their morphology nor by genetic analyses [45] and are therefore termed *Aphanizomenon* sp. in this study.

We used three different strains for the experiments for generalization of observed effects within *Aphanizomenon* sp. from temperate lakes.

### 3.2. Experiments for Testing the Influence of Nitrogen Supply

Batch experiments with each of the three strains were performed both in slightly modified Z8 medium (+N) [46] and in nitrogen-free medium (−N). −N medium was prepared by replacing NaNO_3_ by the equivalent amount of NaCl for maintenance of ion balance. Cultures were started with cell pellets of nutrient saturated pre-cultures which were resuspended with 200 mL of +N or −N medium (Table 1). The cultures grew at 20 °C, light intensity of 105 µE m^−2^∙s^−1^ and continuous shaking at 50 rpm in a 12 h/12 h light-dark cycle. Culture density was determined by measuring the optical density at 750 nm (OD_750_).

### 3.3. Experiments for Testing the Influence of Phosphorus Supply

These experiments were performed using semi-continuous culturing as phosphorus limitation in batch cultures leads to rapid decline of cell density. To obtain the maximum specific growth rates under the given conditions the +P cultures were grown in slightly modified Z8 medium containing 0.172 mM phosphorus and diluted according to the turbidostat principle, maintaining a target culture density of OD_750_ =0.1 after each dilution. For the experiments at defined phosphorus limitation (−P), we diluted cultures following the chemostat principle, *i.e*., by setting a fixed dilution rate of 0.1 per day, which corresponded to just about half of the growth rates attained in the +P experiments. To ensure comparable cell densities in +P and −P settings a reduced phosphorus concentration of 0.034 mM was supplied in the −P medium. Actual growth rates were estimated using the OD_750_ determined before each dilution.

All cultures were run at light intensities of 70 µE m^−2^∙s^−1^ with 12 h/12 h light-dark cycle and continuous shaking at 20 °C. Cyanobacterial suspension (100 mL) was filled into 500 mL-Erlenmeyer flasks and diluted every second day. Cultures were pre-cultured for at least 2 weeks to make sure they are in a steady state with complete adaptation to the given conditions before experiments were started.

### 3.4. Determination of Cyanobacterial Biovolume

Biovolume (BV) was estimated based on previously established relationships (calibration curves) between BV and OD_750_ which resulted from microscopic counting and measuring of at least 10 different samples for each strain.

As OD measurements detect both dead and viable cells and their ratio can vary in batch cultures, the estimated BV was corrected for the share of the dead cells measured via epifluorescence microscopy (see below) for all batch cultures. This was not necessary for the semi-continuous cultures, as under steady state conditions the ratio of dead to viable cells remains constant.

### 3.5. Toxin Analysis

Analyses distinguished between CYN and D-CYN, and their sum is referred to as cylindrospermopsins (CYNs) in this paper.

Cyanobacterial material for analysis of CYNs was harvested from the steady state at 10 successive dilution days from the semi-continuous cultures as well as every other day or every three days from batch cultures. For analysis of cellular CYN contents, samples were filtered over RC55 membrane filters (regenerated cellulose, 0.45 µm pore size, Whatman, Freiburg, Germany). Sample volume was 10–20 mL for the nitrogen experiments (depending on culture density) and 10 mL for the phosphorus experiments. Filters and filtrates for analysis of extracellular CYNs concentrations were stored at −20 °C until analysis. Filters were extracted according to Welker *et al.* [47] by adding 1 mL water, sonication for 10 min and shaking for 1 h. After centrifugation, the extraction was repeated and the pooled supernatants were vacuum-dried. Prior to analysis, the dried extracts were dissolved in 1 mL water.

Analyses for CYN and D-CYN were carried out on an Agilent 2900 series HPLC system (Agilent Technologies, Waldbronn, Germany) coupled to a API 5500 QTrap mass spectrometer (AB Sciex, Framingham, MA, USA) equipped with a turbo-ionspray interface. The extracts were separated using a 5 µm Atlantis C18, 2.1 × 150 mm column (Waters, Darmstadt, Germany) at 30 °C. The mobile phase consisted of water (A) and methanol (B) both containing 0.1 formic acid, and was delivered as a linear gradient from 1%–25% B within 5 min at a flow rate of 0.25 mL min^−1^. The injection volume was 10 µL.

The mass spectrometer was operated in the multiple reaction monitoring mode (MRM). For the determination of CYN, the transitions *m*/*z* 416.1 (M + H^+^) to 194 and 416.1/176, and for D-CYN the transitions *m*/*z* 400.1 (M + H^+^) to 194 and 400.1/320 were monitored with a dwell time of 0.1 s. Quantitation of CYN and D-CYN was performed with the most intensive transitions, *m*/*z* 416.1 (M + H^+^) to 194 and 400.1/194, respectively.

Standard curves were established for CYN (National Research Council, Ottawa, Canada) and D-CYN (Novakits, Nantes, France) and analyzed in line with the unknowns (one calibration curve after 30 unknowns). The detection limit was 0.1 pg on column for CYN and 0.2 pg for D-CYN.

Toxin concentrations were related to the estimated BV on the respective sampling day. In the batch cultures, the continuous release of CYNs leads to an enrichment over time, and this was taken into account by relating the extracellular CYNs not to BV at the time of sampling, but to the area under the BV curve (integral) between start and time points of sampling.

### 3.6. Epifluorescence Microscopy and Staining to Identify Impaired Filaments

Culture suspensions from the batch experiments were stained with SYTOX^®^ Green Nucleic Acid stain (Molecular Probes, Invitrogen, Darmstadt, Germany) for visualization and counting of cyanobacterial cells with permeable membranes. The original SYTOX^®^ solution (5 mM in DMSO) was prepared as working solution by diluting 1:100 (*v*:*v*) with water. 10 µL of that working solution was added to 1 mL cyanobacterial suspension. After 5 min dyeing at room temperature in the dark, the samples were examined by epifluorescence microscopy with a Zeiss Axiovert 100 (Zeiss, Jena, Germany) equipped with a HBO 50/AC mercury light bulb and a filter set containing excitation filter BP 450-490, chromatic beam splitter FT 510 and barrier filter BP 515-565.

Intact biovolume and volume of cells with permeable membranes were determined by using a digital image analysis with IQ EasyMeasure^®^ (INTEQ GmbH Berlin, Berlin, Germany).

## 4. Conclusions

An active release of CYN has been under discussion for some time, based both on the high amounts of extracellular CYN found in previous studies and on the indication of the existence of a transporter in the CYN biosynthesis gene cluster. Furthermore, the biosynthesis of CYN is assumed to depend on nitrogen availability. Our results support both hypotheses—they show that CYN is actively released from intact cells under conditions of nitrogen availability and phosphorus limitation, and that simultaneously the ratio of D-CYN to CYN substantially increased as cultures grew. Cylindrospermopsin synthesis and release therefore seem to be linked to nutrient metabolism. These findings have implications for risk assessment regarding the consequences of population growth and toxicity of *Aphanizomenon* spp. as well as for risk management regarding effective treatment methods for removing cylindrospermopsins from drinking water.
